# Effects of TRAP-1-Like Protein (TLP) Gene on Collagen Synthesis Induced by TGF-β/Smad Signaling in Human Dermal Fibroblasts

**DOI:** 10.1371/journal.pone.0055899

**Published:** 2013-02-13

**Authors:** Xue Wang, Yunliang Qian, Rong Jin, Yan Wo, Jun Chen, Chen Wang, Danru Wang

**Affiliations:** Department of Plastic and Reconstructive Surgery, Shanghai Ninth People’s Hospital, Affiliated to Shanghai Jiao Tong University School of Medicine, Shanghai, People’s Republic of China; Leiden University Medical Center, The Netherlands

## Abstract

**Background:**

Hypertrophic scars are pathologic proliferations of the dermal skin layer resulting from excessive collagen deposition during the healing process of cutaneous wounds. Current research suggests that the TGF-β/Smad signaling pathway is closely associated with normal scar and hypertrophic scar formation. TRAP-1-like protein (TLP), a cytoplasmic protein, has been reported to efficiently regulate Smad2- and Smad3-dependent signal expression in the TGF-β pathway. The relationship between TLP and Type I/III collagen (Col I/III) synthesis explored in the present study provides an effective target for wound healing and gene therapy of hypertrophic scarring.

**Objective:**

To investigate the effects of TLP on collagen synthesis in human dermal fibroblasts**.**

**Methods:**

Lentiviral vectors encoding TLP was constructed to transfect fibroblasts derived from normal human skin. The expression of Col I/III and phosphorylation of Smad2 and Smad3 in fibroblasts were examined after TLP treatment. In addition, the comparison of TLP expression in normal skin tissues and in hypertrophic scar tissues was performed, and the effect of TLP on cell viability was analyzed by MTT assay.

**Results:**

TLP expression in hypertrophic scar tissue was markedly higher than in normal skin tissue. The Real Time PCR and Western blot test results both revealed that the synthesis of Col I/III was positively correlated with the expression of TLP. TLP also facilitate Smad2 phosphorylation while, conversely, inhibiting Smad3 phosphorylation. TLP may play a cooperative role, along with the cytokine TGF-β1, in improving the overall cell viability of skin fibroblasts.

**Conclusions:**

TLP likely acts as a molecular modulator capable of altering the balance of Smad3- and Smad2-dependent signaling through regulation of phosphorylation, thus facilitating collagen synthesis in fibroblasts. Based on genetic variation in TLP levels in different tissues, these results suggest that TLP plays a key role in the process of TGF-β1/Smad3 signaling that contributes to wound healing and genesis of pathologic scars.

## Introduction

Scar, the inevitable complication of wound healing, often incurs excessive proliferation of fibrous tissue with the potential to result in deformity of appearance, paraesthesia, and even organ dysfunctions, leading to significant psychological diseases for burn survivors. Hypertrophic scars may result from abnormal fibrous wound healing that has exhibited reduced or absent tissue repairment and regeneration regulating mechanisms. Resultant imbalance between these factors and subsequent excessive accumulation of collagen may lead to tissue fibrosis, a condition that may enhance production and deposition or, alternatively, impair degradation and removal of collagen. Few effective therapies have been under contemporary research due to the poorly defined mechanism of scar formation [1].

The TGF-β mediated signaling pathway is believed to be closely associated with wound healing and scar formation [2]. Previous researches have shown that TGF-β1, TGF-β receptor types of I and II, and Smad3 are all highly expressed in pathological scar tissue, indicative of a close relationship between TGF-β signal transduction and scar tissue proliferation [3]. Deepened understanding of the TGF-β signal transduction pathway has led increasing investigators to attempt at the inhibition of TGF-β transduction at various levels. Examples of these therapies include treatment with TGF-β antagonists [4], truncated TGF-β1 receptors [5], compounds capable of blocking the Smad3 signaling pathway [6], induced overexpression of Smad7 [7], and glucocorticoids that block intranuclear signals [8]. Though these therapies all have exhibited some degree of definite efficacy, each is inevitably influencing biological effects of other signaling pathways. Some therapies have even been shown to be adverse to wound healing, such as overinhibition of the fibronectin synthesis. These effects have been puzzling investigators over the past decades by suggesting the existence of an undetermined target protein possessing specific and important biological effects on signaling pathways. Efficient and specific downregulation of such a protein could play a significant role in the expression of its downstream signals, thus affecting wound healing and scar formation.

TRAP-1-like protein (TLP), an intermediate protein in TGF-β signaling pathway, is a novel human cytoplasmic protein recently separated and characterized. TRAP-1 is a specific molecular chaperone for Smad4, which brings Smad4 into the vicinity of the receptor complex and facilitates its transfer to the receptor-activated Smad proteins [9]. As a homologue of TRAP-1 with approximately 25% homology, it has been named as TRAP-1-like Protein (TLP), and it is also known as hVPS39 (human vacuolar sorting protein39) and hVam6p(human vesicle associated membrane protein 6). TLP has been found to be associated with the TGF-β/Smad signaling transduction pathway in addition to as a regulator in the transduction pathway, though its most significant role is likely its ability to regulate Smad2/3 in opposite directions simultaneously. Overexpression of TLP suppresses the expression of the Smad3/4 specific receptor protein (SBE) induced by TGF–β. Conversely, abundant TLP may strengthen the expression of the Smad2/4 specific receptor activin response element (ARE) [10].

TLP, a newly discovered molecule with high biological value, still remains unclear as to its relationship with the TGF-β/Smad signal transduction pathway and its role in fibrosis. This study probed into the problem of how TLP affected the TGF-β/Smad signaling pathway, including its effects on the downstream signals and on collagen synthesis, with a view to providing attractive therapeutic targets to interfere with TGF-β signaling in wound healing and preventing hypertrophic scar formation processes.

## Materials and Methods

### Patient Inclusion Criteria

Only virgin mature pathologic scars without prior steroid injection, radiation therapy, and surgical excision were selected. “Mature” was defined as scars over 1 year old in areas under minimal tension with no apparent physical change in scar appearance over at least 6 months [11]. Before surgery, all the patients were informed of the purpose and procedure of this study and agreed to offer their excessive tissue. The written consent was obtained from all participants involved in this study. All the protocols were approved by the Ethic Committee of Shanghai Ninth People’s Hospital affiliated to Shanghai Jiao Tong University School of Medicine.

### Construction of Lentivirus Vectors Containing the TLP cDNA Gene

The target gene, TLP, cDNA was amplified by PCR with TLP-Forward Primer: 5′- GCTCTAGAGCCACCATGCACGACGCTTTCGAGCCAG-3′ and TLP-Reverse Primer: 5′- CGGGATCCTCAAGTGTCAGCTGGGTTTACC -3′. After construction of the TLP gene recombination expression vector, pTLP-GFP lentivector, constructed plasmids were selected for sequencing. The destination plasmid pcDNA-TLP was cotransfected with pPACK™ Lentivector Packaging plasmids into 293TN cells (ATCC, USA)to produce lentiviral vectors by transfection reagent Lipofectmine 2000, and the virus titer was analyzed with the gradient dilution method.

### Cell Culture and Transfection

Experimental samples were collected from patients undergoing operations in the Department of Plastic Surgery, Ninth People’s Hospital affiliated to Shanghai Jiao Tong University School of Medicine. As previously described [12], fibroblasts were obtained from normal human skin after enzymatic digestion. Skin tissue was cut into 0.5 cm3 pieces and the epidermis and dermis were isolated by digestion with 0.25 g/l Dispase II. The dermal tissue was minced and digested thoroughly with 30 volumes of 200 U/ml collagenase I solution at 37°C for 2 hours. Then cells were collected by centrifugation and were further cultured in high glucose Dulbecco’s Modified Eagle Medium (DMEM) plus 10% fetal bovine serum (FBS) under conditions of 5% CO2 at a temperature of 37°C. Cells were passaged 2 to 7 times and used in the following experiments. Cells were subsequently divided into six groups (4 experimental and 2 control groups): Lv-TLP, Lv, control, Lv-TLP-TGF-β1, Lv-TGF-β1, and control-TGF-β1. Following a 72 h incubation period, the infected fibroblasts were observed under fluorescence microscopy, and then were subsequently harvested. Cells in the TGF-β1 cytokine group were seeded into 6-well plates for 24 h, and then were added with TGF-β1 at final concentration of 10 ng/ml after an 8 h starvation period. These cells were subsequently cultured for an additional 24 h under normal conditions. Resultant cell samples were collected for use in the following analyses.

### Real Time-PCR

Total RNA was obtained from normal skin, hypertrophic scar tissues, and normal skin fibroblasts using TRIzol (Invitrogen, Carlsbad, CA, USA) according to the manufacturer’s instructions. RNA concentration and purity was measured by determining the 260/280 nm ratio. All ratios were greater than1.8. First-strand cDNA was synthesized with reverse transcriptase using the total RNA extract. Real-Time polymerase chain reaction (PCR) amplification was performed in triplicate, using the SYBR Green PCR reagent system (SYBR-green; Invitrogen, Carlsbad, CA, USA); Amplification of cDNA fragments and analysis was performed on a thermocycler (iCycler; Bio-Rad, Germany); the methods were as reported previously [13]. Primers used in this section were shown as follows: β-actin: 5′-CCTGTACGCCAACACAGTGC-3′ and 3′- ATACTCCTGCTTGCTGATCC-5′, TLP: 5′-GAAGGCACTCCCACCATC-3′ and 3′-AGCCCCTTCTTCTCATACAG-5′, TGF-β1∶5′-CTGCTACCGCTGCTGTGGCTACTG-3′ and 3′-CGGTCGCGGGTGCTGTTGT-5′, Col I: 5′-ATGTCCACCGAGGCCTCCCAGAAC-3′ and 3′- CCCAGGCTCCGGTGTGACTCGTG-5′, Col III: 5′- CCTGGTCCTTGCTGTGGTGGTGT-3′ and 3′- GCAGTTTCTAGCGGGGTTTTTACG-5′.

### Western Blot

Immunoblotting was performed as described previously [14]. For determination of TLP, Col I/III, and Smad2/3-pSmad2/3, equal amounts of proteins (15 ug) were separated by 10% sodium dodecyl sulfate polyacrylamide gel electrophoresis (SDS-PAGE) and blotted with polyvinylidene fluoride (PVDF) membranes(Milipore, Bedford, MA, USA). After being blocked with 5% defatted milk and washed, membranes were probed with anti-TLP (1∶800, ab90516, rabbit polyclonal, Abcam, Cambridge, UK), anti-TGF-β1 (1∶500, sc-52891, Santa Cruz, California, USA), anti-Col I (1∶1500, C2456, polyclonal, Sigma, St. Louis, MO,USA), anti-Col III (1∶2000, C7805,polyclonal, Sigma, St. Louis, MO,USA), anti-Smad2 (1∶800, SC-101153, Santa Cruz, California, USA), anti-Smad3 (1∶800, sc-101154, Santa Cruz, California, USA), anti-pSmad2 (1∶600, SC-135644, Santa Cruz, California, USA), and anti-pSmad3 (1∶500, sc-130218, Santa Cruz, California, USA) at room temperature for 1 h and then incubated with anti-mouse or anti-rabbit IgG conjugated with horseradish peroxidase. After final treatment with Amersham ECL reagents, samples were exposed to X-ray film for specified time periods in order to detect and record relevant protein bands.

### Cell Viability Assay

A parallel set of plates was assembled, seeded, and exposed as described previously for a microculture tetrazolium (MTT) assay [15]. The absorbance was then measured at 570 nm in a TECAN GENios plate reader.

### Statistical Analysis

The statistical software package SPSS 17.0 was used for analysis. All statistical analysis was performed using the one-way ANOVA with a value of P less than 0.05 or 0.01 considered to represent significant difference (P<0.05 or P<0.01). Data is presented as the mean ± SD of n experiments, as indicated in the figure legends.

## Results

### Construction the TLP Gene Delivery System Mediated by Lentivirus Vectors

**Figure 1:**
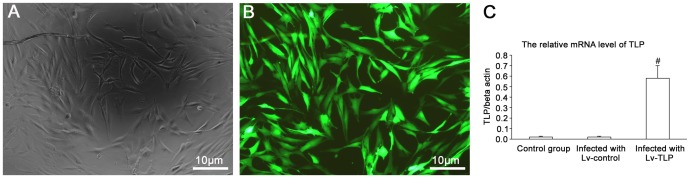
The results of the constructed lentivirus-TLP transfecting human primary skin fibroblasts (HSFs). (**A, B**) HSFs were transfected after 72 h under light and fluorescence microscopy. MOI = 20, 120×. Cells expressed green fluorescent protein (GFP) at 72 h after transfection. The expression of GFP was stable after several passages. (**C**) Real Time-PCR analysis of TLP overexpression in HSFs transfected by Lv-TLP after 72 h. The groups were designed as control group, infected with control lentivirus and infected with recombinant lentivirus (Lv-TLP). The TLP expression in the transfected cells was significantly higher than that observed in control. Results are shown as means ±SD (n = 5) and compared by one-way ANOVA, **^#^**P<0.05.

Constructed plasmids were selected for sequencing, and DNA sequence data was totally aligned with the relevant records in database of the National Center for Biotechnology Information (NCBI). Following stable transfection of human primary skin fibroblasts (HSFs) with Lv-TLP, more than 90% of HSFs samples presented green fluorescence (**Figure 1A, 1B**), indicating that the vast majority of these cells had been successfully transfected with TLP. These results were validated by fluorescence microscopy at 72 h post transfection. Real-Time PCR results further indicated that the HSFs samples infected by Lv-TLP expressed high levels of TLP mRNA in contrast to both the HSFs samples transduced with Lv-GFP and the control groups that did not undergo vector treatment (**Figure1C**). The resultant TLP overexpression model of mammalian skin fibroblasts mediated by lentivirus was thus successfully confirmed.

### Detection of TLP Gene Expression and its Influence on the Synthesis of Col I/III

**Figure 2:**
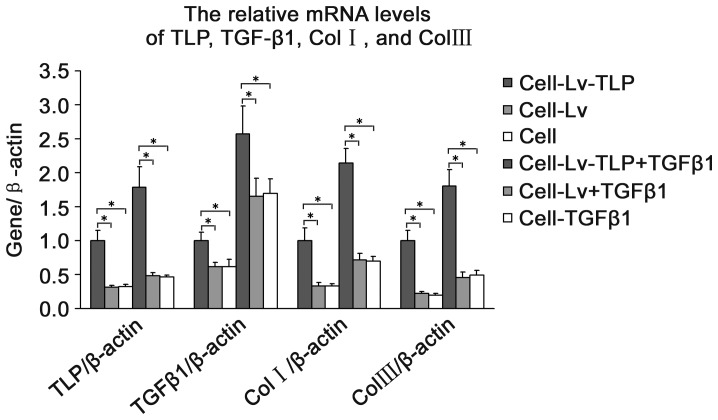
The mRNA levels changes of TGF-β1, Col I, and Col III after TLP treatment. The groups were designed as Cell-Lv-TLP (HSFs transfected with recombinant lentivirus), Cell-Lv (HSFs transfected with control lentivirus ), Cell (HSFs without any treatment), Cell-Lv-TLP+TGF-β1 (HSFs transfected with recombinant lentivirus and stimulated by the cytokine TGF-β1), Cell-Lv+TGF-β1 (HSFs transfected with control lentivirus and stimulated by TGF-β1), and Cell+TGF-β1 (HSFs stimulated by TGF-β1 ). After RNA isolation and reverse transcription, TGF-β1 mRNA was quantified by quantitative PCR, data represents mean±SD, n = 5. * means P<0.05 vs. the control groups with or without TGF-β1.

**Figure 3:**
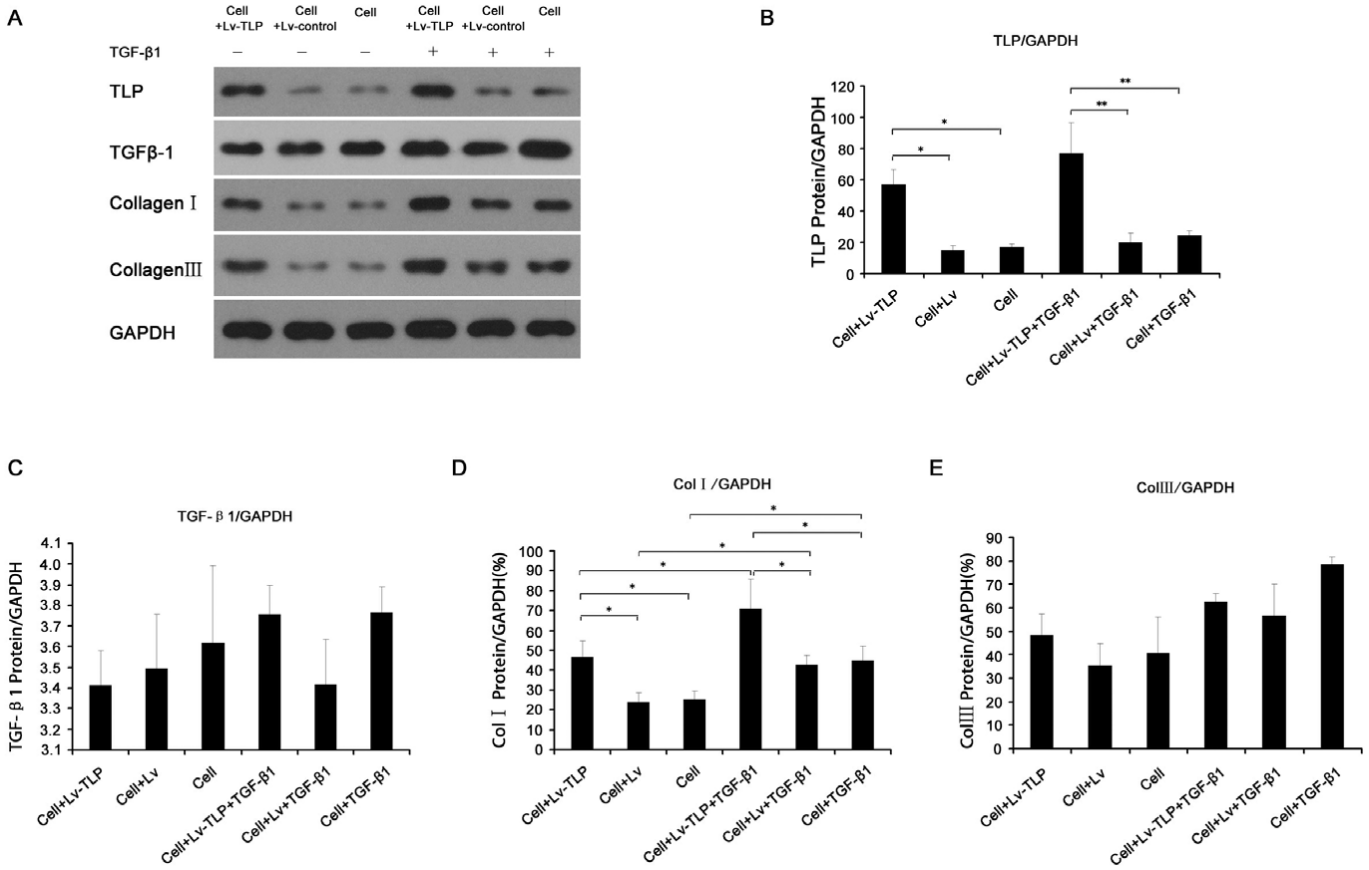
Detection of TLP gene expression and its influence on the synthesis of Col I/III protein. (**A**) Immunoblot analysis of lysate samples for TLP, TGF-β1, Col I, and Col III. (**B, C, D, E**) Determination of grey value of TLP, TGF-β1, Col I, and Col III revealed in **A**. Experiments were repeated 3 times, and data are expressed mean±SD, N = 3. * means P<0.05 between the two groups.

Six groups underwent TLP and Col I/III gene expression analysis: Lv-TLP, Lv, control, Lv-TLP-TGF-β1, Lv-TGF-β1, and control-TGF-β1. As hypertrophic scarring is characterized by overabundant collagen synthesis, analysis of collagen of type I and III gene transcription and protein expression levels was completed using Real-Time PCR and Western blot after 72 h of TLP treatment. As shown in **Figure 2**, the expression of Col I/III in the high TLP expression group was significantly elevated above levels observed in control groups (P<0.05), up-regulated more than 1.5 times and 2 times respectively. Furthermore, with cytokine TGF-β1 stimulation, the amount of the synthesized collagen in the high TLP expression group was also markedly increased than the control groups (P<0.05). Protein level analysis also showed the similar tendency towards to Figure 2 but except collagen III. As shown in Figure 3E, although there was no statistical significance existing between groups, among all the panel experiments under same conditions we obtained the consistent results that the Col III expression were about enhanced by 10–15% after TLP treatment.While the variation were not so apparent under TGF-β1 stimulation. Notably, the amount of the expressive TGF-β1 appeared constant (Figure 3C).

### Alteration of the Expression of p-Smad2 and p-Smad3 Affected by TLP

**Figure 4:**
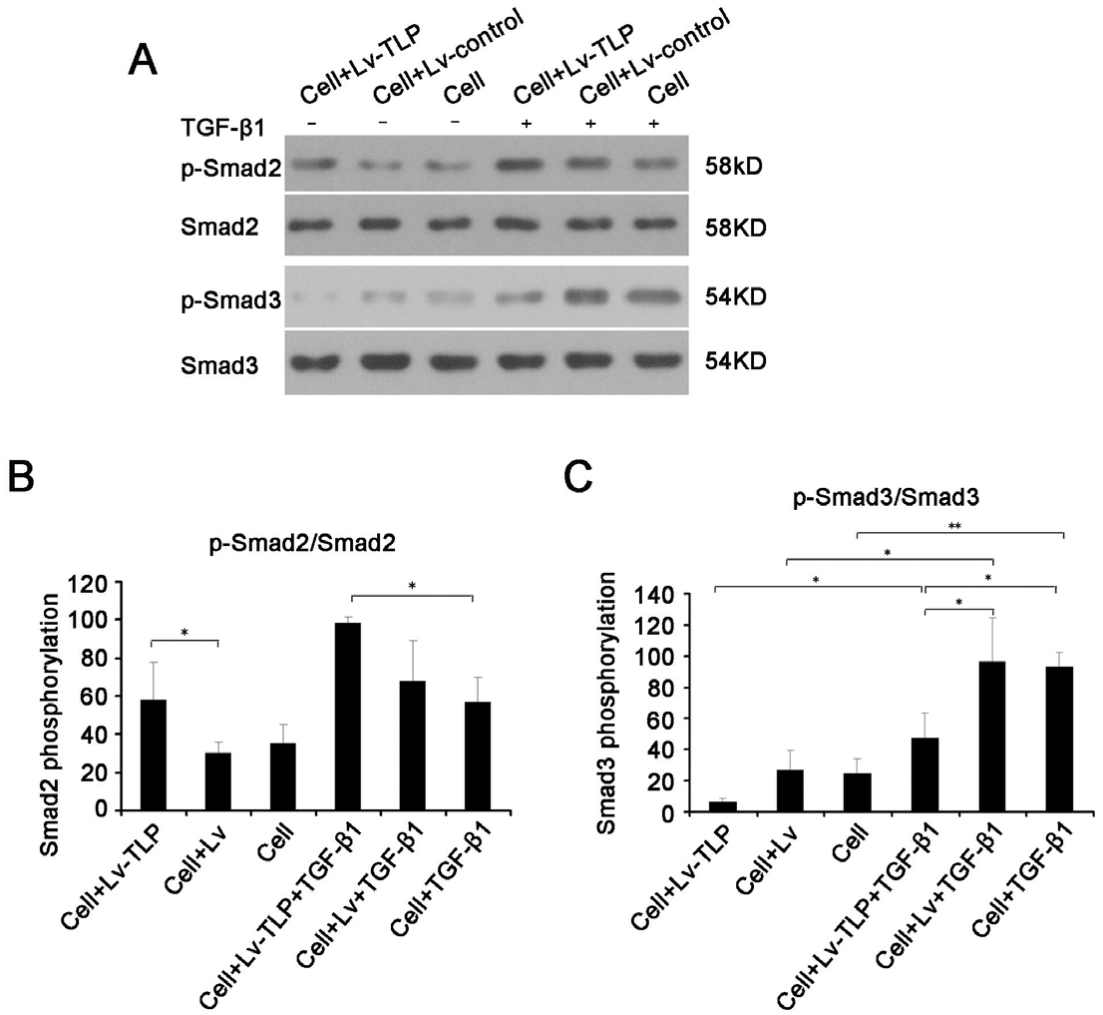
Western blot analysis demonstrates that TGF-β/Smad signaling changes after TLP overexpression. (**A**) The changes in phosphorylation of Smad2 and Smad3. (**B, C**) Determination of grey value of pSmad2/Smad2 and pSmad3/Smad3. Results were shown as mean±SD of gray value. * means P<0.05 and ** means P<0.01 between two groups.

The intrinsic mechanism of alteration in collagen expression triggered by TLP is further revealed by examining the expression levels of Smad2/P-Smad2 and Smad3/P-Smad3 (shown in **Figure 4**). Under conditions of high TLP expression, the level of p-Smad3 decreased approximately by 25% (P<0.05). Conversely, the level of p-Smad2 clearly increased by more than 20% (P<0.05). Furthermore, with treatment of TGF-β1, the similar variations were found among the experimental groups.

### The Differential Expression of TLP and the Associated Molecules between Hypertrophic Scars and Normal Skin Tissues

**Figure 5:**
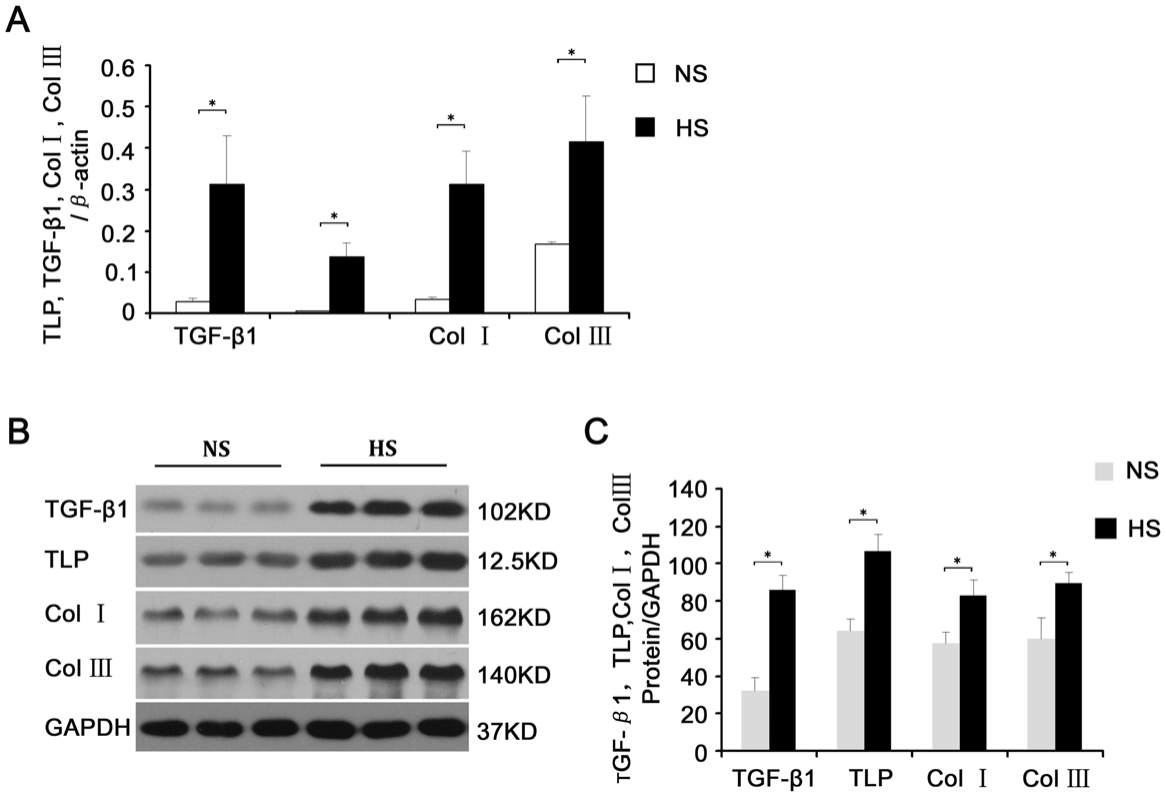
The differential expression of TLP and the associated molecules between hypertrophic scars and normal skin tissues. Samples proteins were respectively extracted from three different patients’ skin tissue and another three patients’ hypertrophic scar, which were harvested with the same criteria and no history of keloid. (**A**) Comparison of transcription levels of TLP, TGF-β1, Col I, and Col III in hypertrophic scar versus normal skin tissues. (**B**) Western blot analysis of variation between TLP, TGF-β1, Col I, and Col III expression in hypertrophic scar versus normal skin tissues. (**C**) Determination of grey value of TLP, TGF-β1, Col I, and Col III from hypertrophic scar and skin tissues. Results were shown as mean±SD of gray value. * means P<0.05 between hypertrophic scar and normal skin tissues. The representative analyses of 3 experiments were shown.

The TLP mRNA levels in hypertrophic scar tissues were 15 folders higher **(** **Figure 5A** **)** than in normal skin, and higher by up to 80% in the protein level **(** **Figure 5B, 5C** **)**. In concurrence with previous reports, the expression levels of Col I/III and TGF-β in hypertrophic scars were also markedly higher than those observed in normal skin samples both mRNA and protein levels(shown in **Figure 5**).

### MTT Assay

**Figure 6:**
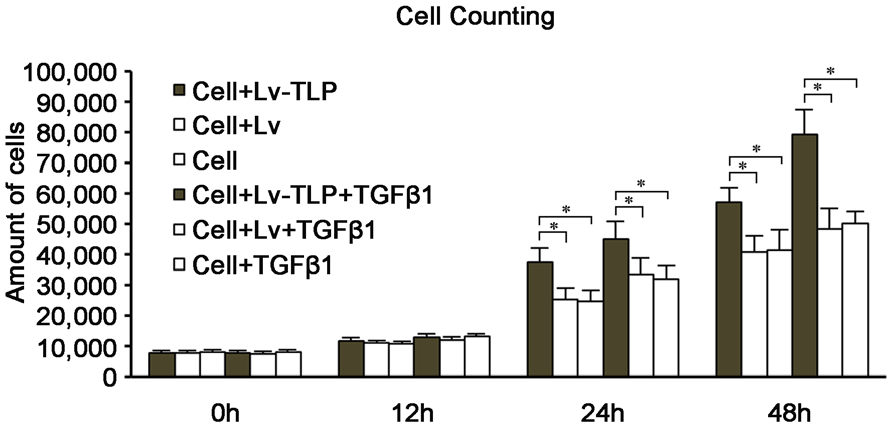
Variabilities of cell proliferation in different group over time. The amount of HSFs were counted at the time points of 0 h, 12 h, 24 h, 48 h after being seeded into 96 plates. Values were expressed as the mean±SD (n = 5) P<0.05 compared to the groups of cell and cell-TGF-β1 using one-way ANOVA.

To determine the effect of TLP on the cell viability, MTT assays were performed. The results showed that before 12 h after TLP treatment, there was no obvious statistical difference among all groups. However, when at 24 h cell viability was increased by up to 40% (**Figure 6**). Upon treatment with TGF-β1, variation within cell viability became more apparent among the experimental groups. Thus, TLP may act cooperatively with TGF-β1 to increase cell proliferative viability. Moreover, compared to control cells, the lentivirus transfected cells showed no sign of cytotoxicity and weak multiplication capacity, that is to say, the gene delivery vector was safe and showed no conspicuous effect on cells.

## Discussion

The biological basis of pathological scar tissue formation is comprised of three closely associated processes, sustained vigorous proliferation of fibroblasts after epithelialization of wounds relative to apoptosis inhibition, disbalances in synthesis and degradation of the primarily collagen extracellular matrix, and abundant supply and prolonged existence of specific growth factors [16], [17], [18]. Additionally, the TGF-β signaling pathway plays an important role in each of these processes. The TGF-β1 signaling mechanism functions through the TGF-β type I (TβRI) and TGF-β type II (TβRII) transmembrane serine/threonine protein kinase receptors. Upon TGF-β1 binding to its type II receptor directly, TβRI is recruited to TβRII where it forms a ligand-receptor heterotetrameric complex [19], [20]. Under physiological conditions, TLP binds the type II receptor even when the pathway has been previously activated by TGF-β1, and the type II receptor is constitutively active. It transphosphorylates and activates the type I receptor, whose direct substrates are Smad2 and Smad3. Phosphorylation of receptor-activated Smads (R-Smads) leads to the formation of complexes with the common mediator Smad (Co-Smad), which are then imported to the nucleus. Nuclear Smad oligomers bind to DNA and associate with transcription factors to regulate expression of target genes [21], [22]. In the process of tissue fibrosis, TGF-β1 is likely to facilitate the expression of the extracellular matrix gene to increase the synthesis and deposition of collagen, fibronectin, and proteoglycan [23], [24]. While, simultaneously, decreasing the yield of cathepsin and enhancing the synthesis of cathepsin inhibitors. In addition, TGF-β1 may strengthen the intercellular adhesion by increasing integrin levels in the extracellular matrix [2]. In the present study, TGF-β1 treatment was shown to increase the phosphorylation levels of Smad2 and Smad3, confirmed by the enhancement of the transcription and expression of collagen mRNA shown in Fig. 3,4,5. Additional confirmation is provided by MTT assay, clearly demonstrating improved cell viability stimulated by TGF-β1 treatment.

In this study, dramatically high expression of Col I/III in the fibroblasts from the group of TLP overexpression was detected not only at mRNA level but also at the protein level **(** **Figure 2** **–** **3** **)**. Tendency exhibiting these variations were very constant no matter cells were stimulated with TGF-β1 or not. In mammalian tissues, we found for the first time that TLP expression in hypertrophic scar tissue is much higher than in normal skin tissue, so do the Col I/III and TGF-β1 **(** **Figure 5** **)**. Thus, this finding further confirms the positive relationship between TLP and collagen synthesis.

The TGF-β/Smad pathway is one of many TGF-β induced pathways, but an increasing number of reports have revealed that Smad3 is required for many cellular responses to injury and disease pathogenesis. Shu-Jen Chen *et al.* found that following transient overexpression of Smad3 and Smad4 in primary human skin fibroblasts, the activation of the α2 (I) procollagen promoter was enhanced. Furthermore, the opposite result was observed in transfected mutant Smad3 demonstrating that Smad3 transmits TGF-β signals from the receptor to the Col I α2 promoter in human fibroblasts, and it is likely to play an important role in stimulation of the ColIα2 promoter activity elicited by TGF-β. In fibroblasts, Smads appear to function as inducible DNA-binding transcription factors [25], as confirmed by the research conducted by Zimin Wang *et al.* wherein suppression of Smad3 expression in human keloid fibroblasts by RNA interface (RNAi) technology revealed that, in comparison with the control, mRNA levels of types I and III proCollagen were also significantly and uniquely decreased following reduction of Smad3 by siRNA [26]. Furthermore, primary hepatic stellate cells exhibiting overexpression of Smad3 showed increased deposition of fibronectin and type I collagen, thus increasing rates of chemotaxis [27]. Direct evidence supporting the involvement of Smad3 in fibrosis is provided by the use of mice with a targeted deletion of Smad3 [28], wherein the Smad3 knockout animal model obviously inhibits Smad3’s facilitation of TGF-β [29], [30]. These various experimental approaches demonstrate the direct implication of Smad3 activation on downstream TGF-β in the pathogenesis of pulmonary fibrosis. However, Smad2-dependent pathway also, to some degree, attributes to the extracellular matrix protein synthesis such as collagens, fibronectin. Previous studies reported that Smad2 inhibition by siRNA significantly downregulated synthesis of fibronectin and collagen type III in TGF-β1-stimulated cells [31]. In the cardiac fibroblasts, the medicine efonidipine could inhibit TGF-β1–induced Smad2 phosphorylation markedly attenuated synthesis of collagens resulting in cardiac fibrosis [32]. Moreover, the down-regulation of Smad2 expression in keloid fibroblasts can significantly decrease procollagen gene expression. Also, siRNA targeting Smad2 was an efficient reagent with which to reduce extracellular matrix deposition and attenuate process of fibrosis. In a word, Smad2 and Smad3 could both be effective therapeutic approaches for improving skin wound healing and inhibiting progression of fibrotic conditions by interrupting the TGF-β signaling pathway.

In this research, up-regulation of exogenous TLP in normal human skin fibroblasts was conducted in the current study and analyzed by Western Blot analysis, detecting that phosphorylation levels of Smad3 decreased by 15%, though phosphorylation levels of Smad2, conversely, increased by up to 25%. And even the variation observed in p-Smad2 levels was much greater than that observed in p-Smad3 levels. However, Angelina’s finding reveals that without affecting the phosphorylation of Smad2 and Smad3, TLP may inhibit the formation of the Smad3/4 complex so as to specifically block the downstream pathway of Smad3 and to up-regulate the Smad2 pathway. Based on this conclusion, it is logical that TLP blocks the Smad3 signaling pathway, thus suppressing the synthesis of Col I/III indirectly. However, the current experiment demonstrates opposite results, drawing doubt upon this conclusion. Based on the findings of the current study, it is more likely that TLP up-regulated the synthesis of collagens mainly through activation of Smad2, perhaps, with other signal pathways through cross-talks or due to the existence of a mutual regulator of many signaling pathways. This may be supported by the finding that the variation observed in p-Smad2 levels was much greater than that was observed in p-Smad3 levels caused by TLP treatment. Smad3 is required for many of the cellular responses to injury and disease pathogenesis, one of which is the Smad3 facilitation of collagen synthesis.

Because the upstream promoters of the 265 extracellular matrix genes have been confirmed to be induced by TGF-β/Smad3-dependent signaling pathways, this hypothesis seems probable [33]. In addition, collagen synthesis has been demonstrated to be affected by many diverse factors. Hence, the action of TLP in these signaling pathways is not simply linear, which might be due to the existence of cross-talks with many other pathways or due to the existence of a mutual regulator of many signaling pathways. It is known that many regulators exist for collagen synthesis, such as type I collagen. Some relevant positive and negative regulatory proteins have also been identified that affect each pathway. To make the matter more complex, there also exist mutual influences and competitions between different pathways that must be considered in living tissues. So far, the undergoing mechanism of TLP still need to further study. TLP represents a novel cytokine only recently discovered and characterize that may play a significant inhibitory role in the Smad3 pathway and activate other cytokines or signaling pathways for the promotion of collagen synthesis. TLP facilitation of the fibrosis process, though indirect, is certainly significant. Although the specific mechanism of the TLP signaling pathway is currently unresolved and pending further investigation, the hypothesis that TLP may act as a regulator to balance the flux of Smad2 and Smad3 in TGF-β signaling and indirectly affect collagens synthesis is worthy of consideration.

The specific mechanism of TLP’s regulating action remains unclear. In 2003, Angelina first reported that TLP can modulate the balance of the Smad2 and Smad3 signal reaction as an intermediate protein molecule in the TGF-β signaling pathway, though the hypothesis provided for the molecular mechanism of TLP’s action lacked support. As early as in 2001, Steve Caplan found that as a mammalian tethering/docking factor, TLP was characterized with intrinsic ability to promote lysosome fusion *in vivo* [34]. In the TLP gene knockout zebrafish model, many syndromes were observed, including notable defects of pigmentation in the retina, skin, and intestine; vision obstruction; defects of visceral function; and defects in the innate immune system. These conditions may be stimulated by the influence of TLP on the transport of endosomal vesicles [35]. Similarly, in the TLP knockout mice model, mouse embryos were found dead in 6.5 weeks, demonstrating the importance of TLP for early embryonic development [36]. As additional research information on TLP became available, researchers moved from the examination of microorganism models to current animal models, including mammalian tissues. Research initiated by cell biology experiments that first identified TLP have progressed to an exploratory explanation for pathogenic genes and embryogenesis. With increasing knowledge of TLP function, its value as a research and clinical target are becoming increasingly apparent.

The physiological effect of TLP overexpression in human primary skin fibroblasts has been initially documented over the course of the current study, demonstrating the essential role of the TLP gene in the process of collagen synthesis and modulation of phosphorylation in both Smad2 and Smad3. Though the intrinsic mechanism of TLP action requires further study, it is speculated that TLP functions during the process of wound healing and tissue fibrosis by acting upon TGF-β signaling modulators.
